# Perforated Hemorrhagic Cholecystitis After Coronavirus Disease 2019 (COVID-19) in a Patient Receiving Anticoagulant Therapy

**DOI:** 10.7759/cureus.89306

**Published:** 2025-08-03

**Authors:** Rintaro Kinjo, Masao Uemura, Takafumi Ihashi, Shigeyoshi Yamanaga, Hiroshi Yokomizo

**Affiliations:** 1 Department of Surgery, Japanese Red Cross Kumamoto Hospital, Kumamoto, JPN

**Keywords:** anticoagulant therapy, covid-19, gallbladder, hemoperitoneum, hemorrhage, hemorrhagic cholecystitis, laparoscopic cholecystectomy, steroid treatment

## Abstract

Hemorrhagic cholecystitis (HC) is a rare but life-threatening condition. While anticoagulant therapy is a known risk factor, the coronavirus disease 2019 (COVID-19) has recently emerged as another trigger. We experienced a severe case of perforated HC complicated by hemoperitoneum in a patient presenting both risk factors. An 87-year-old male patient on apixaban therapy was admitted to a referral hospital seven days before COVID-19, where treatment with antiviral medication and steroids was initiated. Five days before transfer, the patient developed abdominal pain with elevated levels of inflammatory markers. On the day of presentation, the patient experienced sudden right upper abdominal pain and was transferred to our hospital following plain computed tomography (CT), which revealed gallbladder enlargement and suspected hemoperitoneum. Upon arrival, the patient exhibited diffuse abdominal tenderness. Contrast-enhanced CT revealed extravasation from the cystic artery and hematomas around the gallbladder, confirming the diagnosis of HC with hemoperitoneum. Emergency laparoscopic cholecystectomy was performed after administration of recombinant Factor Xa (used to reverse anticoagulation). Intraoperatively, hemorrhagic ascites were observed, with the gallbladder appearing tense, gangrenous, and perforated. A histopathological examination confirmed acute cholecystitis with necrosis and perforation. The postoperative course was uneventful, and apixaban therapy was restarted on postoperative day 1. The patient was discharged to a rehabilitation facility on postoperative day 13. Emergency laparoscopic cholecystectomy was lifesaving in this patient with HC and hemoperitoneum.

## Introduction

Hemorrhagic cholecystitis (HC), although rare, is a severe variant of acute cholecystitis, accounting for approximately 0.55% of acute cholecystitis cases, with a reported mortality rate of 15%-20% [1]. Risk factors include cholelithiasis, vascular abnormalities, trauma, iatrogenic factors, neoplasms, and anticoagulant therapy [2]. Notably, the increasing global use of anticoagulants such as direct oral anticoagulants (DOACs) appears to contribute to the rising risk of HC [3]. In addition, coronavirus disease 2019 (COVID-19) has recently emerged as a potential trigger for HC. Herein, we report a particularly severe case of perforated HC that progressed to hemoperitoneum in a patient with concurrent COVID-19 and anticoagulation therapy.

## Case presentation

**Figure 1:**
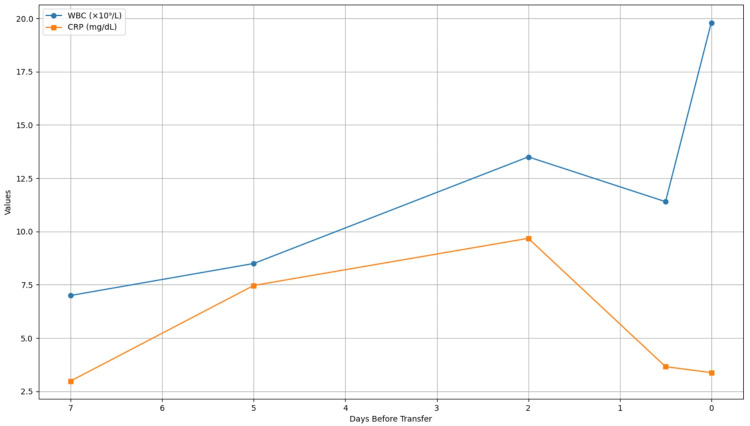
Trend in inflammatory markers (WBC and CRP), as observed from the referral hospital to our facility The patient's inflammatory markers demonstrated a dynamic trend during his clinical course. At the referral hospital, both WBC count and CRP levels progressively increased over several days, with WBC rising from 7 × 10⁹/L to 13.5 × 10⁹/L and CRP from 2.99 mg/dL to a peak of 9.68 mg/dL. This trend was initially interpreted as pyelonephritis and managed with antibiotics. Although markers began to decline after escalation to meropenem, WBC elevated to 19.8 × 10⁹/L on admission to our facility WBC: white blood cell; CRP: C-reactive protein

An 87-year-old male patient with a history of chronic atrial fibrillation and cardioembolic stroke was transferred to our hospital, complaining of upper abdominal pain that had persisted for five days. The patient had been taking apixaban (5 mg) and limaprost alfadex (30 mg) for more than 10 years. Seven days before transfer, he was diagnosed with COVID-19 and admitted to a referral hospital, where he received remdesivir and dexamethasone for five days. After his COVID-19 symptoms were resolved, he began experiencing abdominal discomfort, accompanied by an elevated white blood cell (WBC) count of 8.5 × 10⁹/L and a C-reactive protein (CRP) level of 7.47 mg/dL (Figure 1). At that time, the patient was clinically diagnosed with pyelonephritis and was treated with ceftriaxone. However, his inflammatory marker levels remained abnormal (WBC of 13.5, CRP of 9.68), prompting a switch to meropenem on two days before transfer (Figure 1). Although his inflammatory marker levels subsequently improved in the morning of the transferred day (WBC of 11.4, CRP of 3.66), he suddenly developed right upper quadrant abdominal pain. Plain computed tomography (CT) scan at the referring hospital revealed a distended gallbladder filled with intraluminal hyperdense fluid and a suspected hemoperitoneum. Based on these image findings and the acute abdominal symptoms, he was transferred to our facility for further evaluation and potential surgical intervention.

Upon arrival, the patient exhibited diffuse abdominal tenderness. His vital signs were as follows: body temperature: 37.7℃, blood pressure: 110/90 mmHg, and heart rate: 130 beats/minute (irregular). Laboratory tests showed WBC of 19,845/μL and CRP of 3.38 mg/dL (Figure 1 and Table 1).

**Table 1:** Laboratory results at our facility after transfer WBC: white blood cell; HGB: hemoglobin; HCT: hematocrit; T-Bil: total bilirubin; AST: aspartate aminotransferase; ALT: alanine aminotransferase; ALP: alkaline phosphatase; CRP: C-reactive protein

Test name	Result	Normal range	Units
WBC	19.8	4.0-11.0	×10⁹/L
HGB	14.9	13.5-17.5	g/dL
HCT	45.1	38.8-50.0	%
Platelet	170	150-450	×10⁹/L
T-Bil	0.9	0.1-1.2	mg/dL
AST	31	10-40	U/L
ALT	40	7-56	U/L
ALP	74	44-147	U/L
CRP	3.38	<0.3	mg/dL

**Figure 2:**
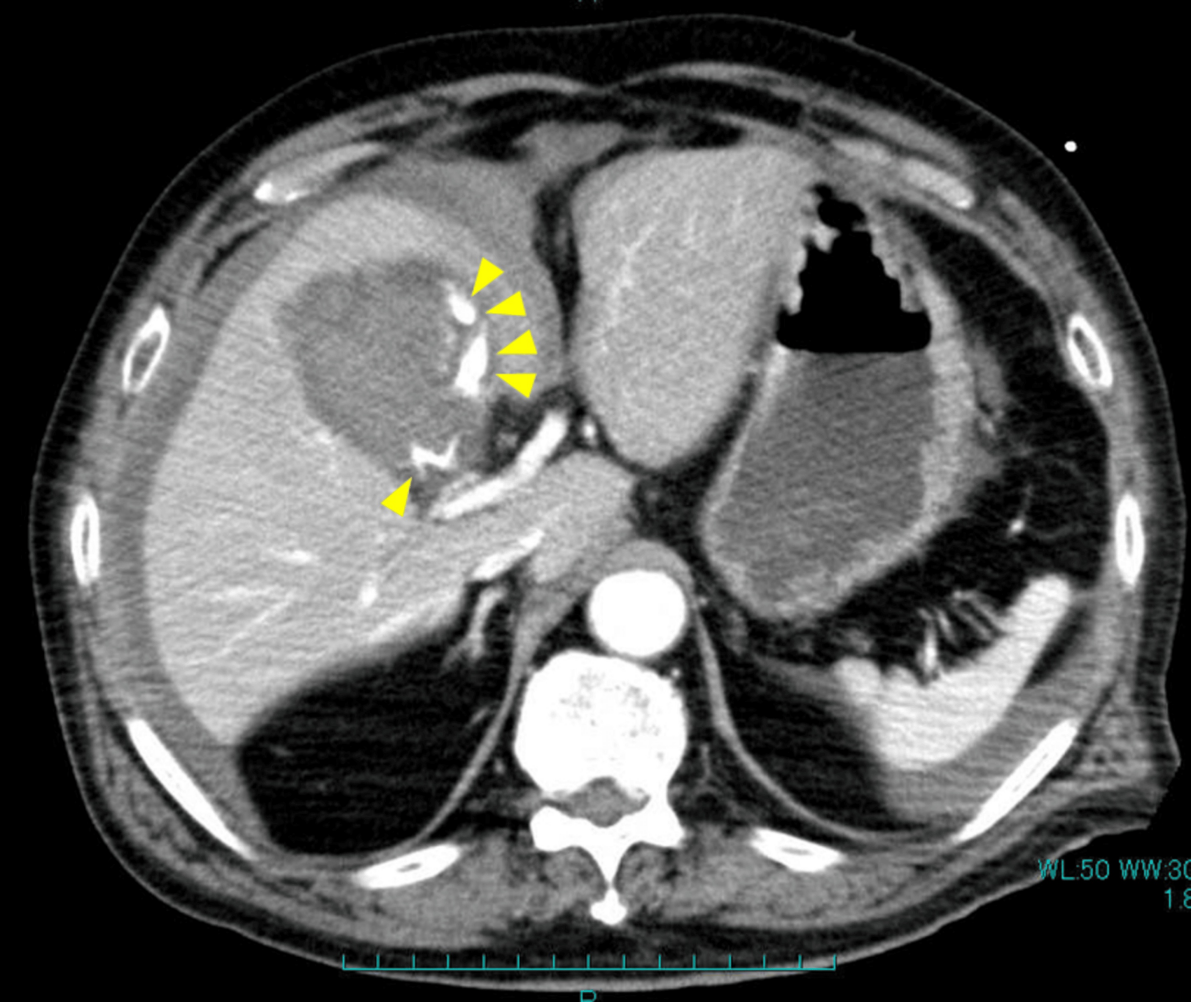
Axial abdominal contrast-enhanced CT scan This image shows gallbladder enlargement with intraluminal hyperdense fluid accumulation and contrast agent extravasation from the cystic artery (arrowheads). Additionally, high-density ascites was observed around the gallbladder, liver, and spleen, raising the suspicion of hemoperitoneum CT: computed tomography

There was no evidence of anemia, coagulation abnormalities, or obstructive jaundice. Contrast-enhanced CT scan revealed extravasation from the cystic artery into the gallbladder along with hyperdense ascites surrounding the gallbladder, liver, and spleen (Figure 2). He was diagnosed with HC and underwent emergency laparoscopic cholecystectomy after the administration of andexanet alfa, a recombinant Factor Xa inhibitor reversal agent.

**Figure 3:**
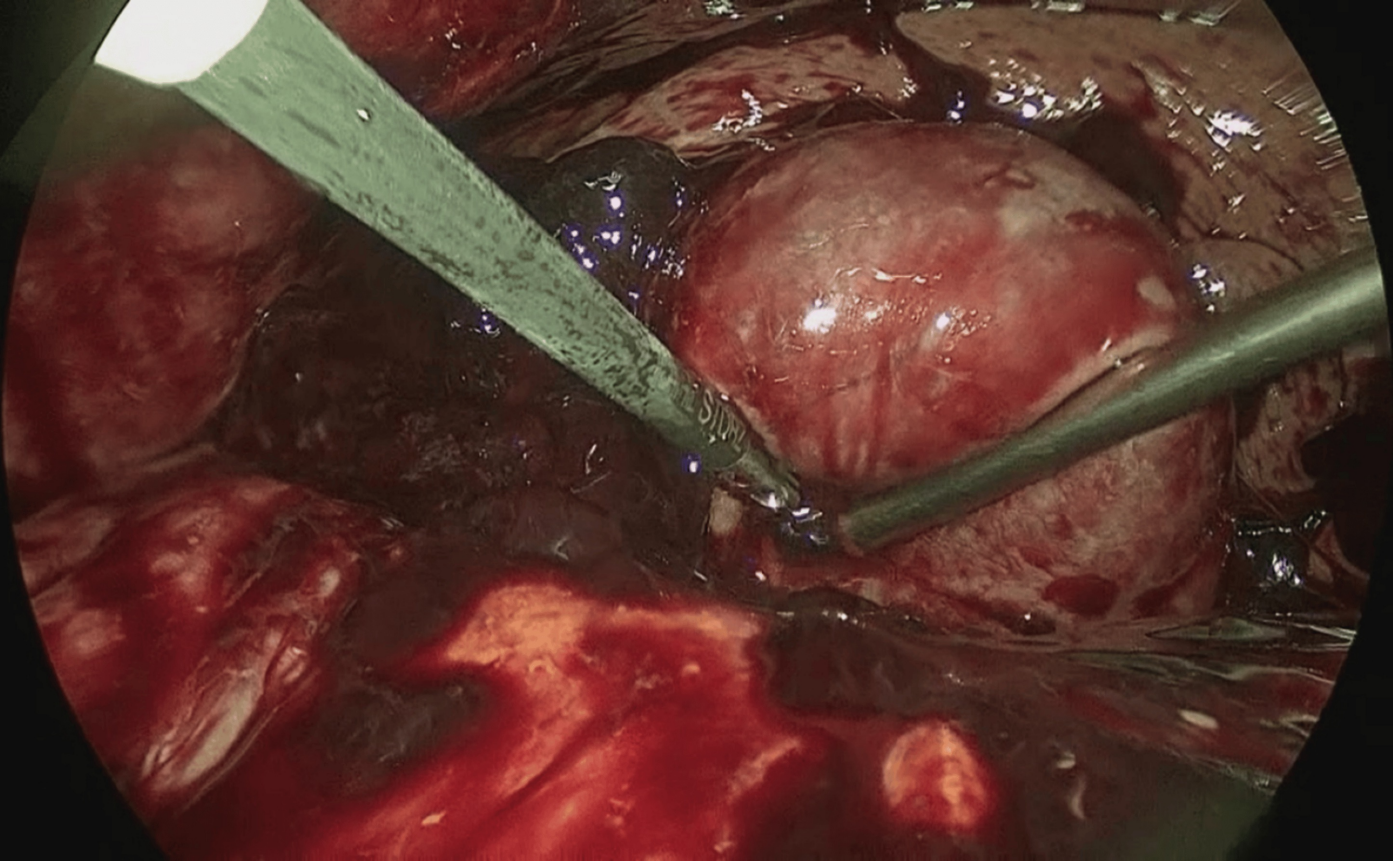
Intraoperative finding Clots and hemoperitoneum were predominantly located in the abdominal cavity. Over 1,100 mL of coagulated hemorrhagic ascites was encountered. The gallbladder appeared tense and dark red, with full-thickness necrosis and perforation on the side of the liver. The gallbladder was extremely fragile and friable. Clots filled the gallbladder, and no stones were identified

During surgery, more than 1,100 mL of coagulated hemorrhagic ascites was encountered, predominantly localized around the liver. The gallbladder was markedly distended and found to be perforated on its right side (Figure 3). The gallbladder was successfully removed with an operative time of one hour and 50 minutes. The total estimated blood loss was 1,200 mL, most of which corresponded to the volume of hemorrhagic ascites. Six units of packed red blood cells and fresh frozen plasma were transfused perioperatively.

**Figure 4:**
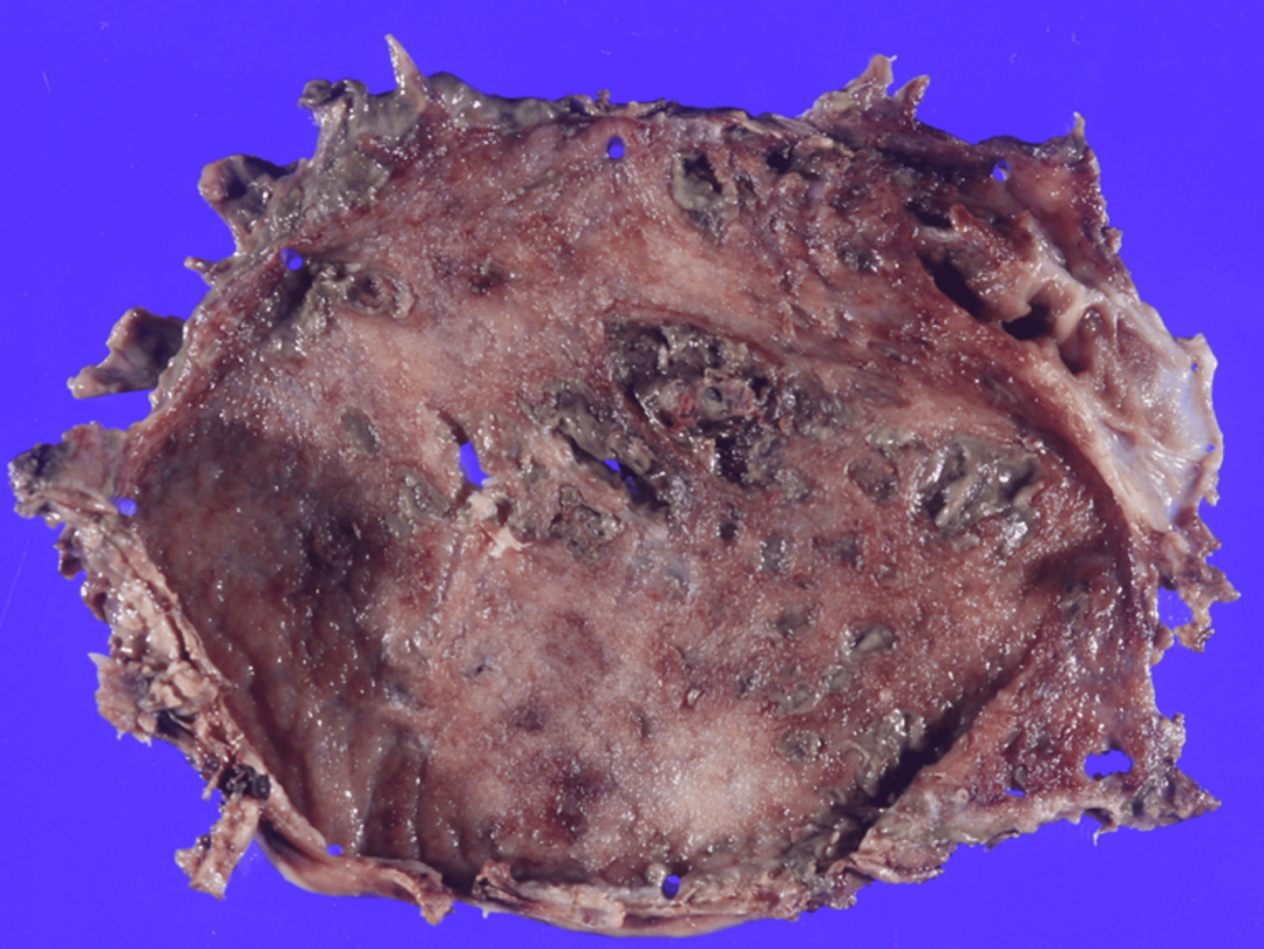
Gallbladder specimen The gallbladder specimen revealed full-thickness necrosis and perforation without wall thickening observed

**Figure 5:**
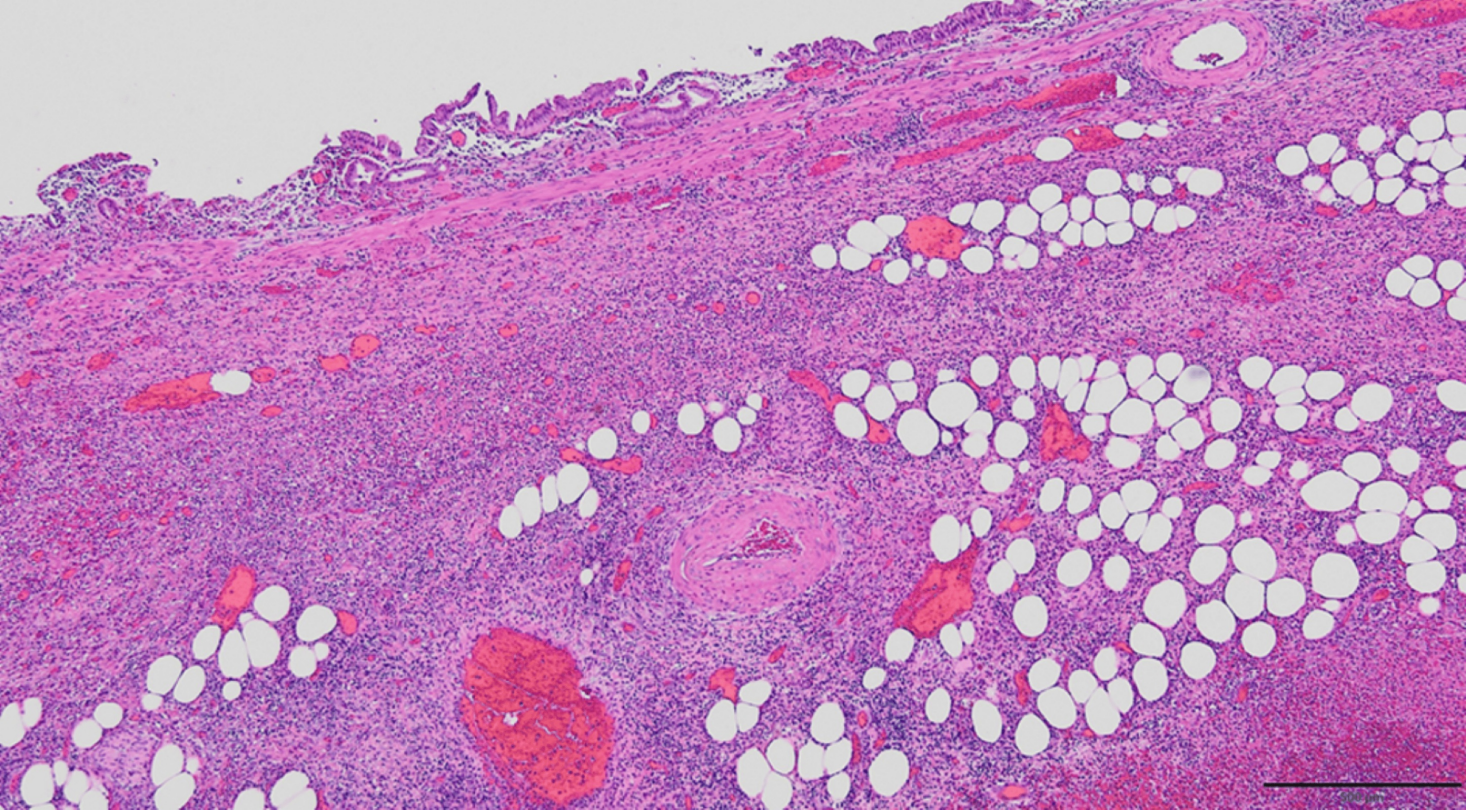
Microscopic image (hematoxylin and eosin stain) of the excised gallbladder Marked infiltration of neutrophils within the wall, along with gangrenous necrosis and perforation, was observed. No granulomas, neoplastic lesions, or vascular anomalies such as aneurysms were present, and the source of the bleeding could not be identified

The gallbladder specimen contained blood clots but showed no evidence of gallstones. Histopathological examination revealed epithelial erosion, regenerative changes, and marked inflammatory cell infiltration within the gallbladder wall, consistent with acute cholecystitis with necrosis and perforation (Figures 4, 5). No granulomas, neoplastic lesions, or vascular anomalies such as pseudoaneurysms were identified. The precise site of bleeding could not be determined.

The patient’s postoperative course was uneventful. Apixaban therapy was resumed on postoperative day 1, given the patient's underlying risk for thromboembolism and clinical stability, and the patient was discharged to a rehabilitation facility on postoperative day 13. The patient was followed up at our outpatient clinic one month after surgery. The patient had no symptoms, and laboratory tests showed no abnormal findings. No imaging studies were performed at that time. Two months postoperatively, additional labs conducted at another facility also remained within normal limits. There were no reported complications related to resumed anticoagulation therapy or residual effects from COVID-19 during this follow-up period.

In summary, the diagnosis of HC in this case was based on several key features. The patient on anticoagulants presented with persistent right upper quadrant abdominal pain following recent COVID-19 and treatment with corticosteroid therapy. Laboratory tests showed elevated inflammatory markers, including a WBC count of 19,845/μL and a CRP level of 3.38 mg/dL. Contrast-enhanced CT demonstrated gallbladder distension, contrast extravasation from the cystic artery, and hyperdense ascites consistent with hemoperitoneum. Intraoperative findings confirmed gallbladder perforation with hemorrhagic ascites. The absence of gallstones and histopathological evidence of necrotic inflammation supported the diagnosis of acute acalculous HC.

## Discussion

The patient, who was receiving apixaban and steroid therapy for COVID-19, developed a perforated HC complicated by hemoperitoneum and underwent an emergency laparoscopic cholecystectomy that saved his life. The incidence of HC is expected to increase with the increase in anticoagulant therapy use over the past decade [3]. Recent cases of HC following COVID-19 treatment have been reported, suggesting that COVID-19 may represent another trigger [4-6].

HC can present in various forms that extend beyond the typical features of acute cholecystitis. Moreover, signs of hemorrhage, such as hypotension, lethargy, and anemia, are observed in only 21% of patients with HC [7]. In subacute cases, blood clots may occasionally obstruct the common bile duct, resulting in jaundice. Blood entering the gastrointestinal tract via the bile duct may manifest as hematemesis or melena. If the gallbladder becomes distended with blood and perforates the peritoneal cavity, it can result in hemoperitoneum. Contrast-enhanced CT scan is considered the optimal imaging modality for identifying a wide range of findings in HC, including active intraluminal contrast extravasation, pseudoaneurysm of the cystic artery, blood clots within the gallbladder, heterogeneous intraluminal fluid, and hemoperitoneum [1,8,9]. However, another review reported that only one of 11 cases with contrast-enhanced CT scans demonstrated hemoperitoneum [7]. The patient presented with fever, signs of peritoneal irritation, and abdominal pain, but no signs of jaundice or gastrointestinal bleeding. CT scan revealed a massively distended gallbladder with active hemorrhage and hemoperitoneum, rare and critical findings in patients with HC.

The causes of HC include trauma, iatrogenic factors, ischemic necrosis due to arteriosclerosis of the cystic artery, inflammation, tumors, ulcers, stones, dialysis, anticoagulant therapy, hemophilia, collagen diseases, and other conditions [10]. The mechanisms underlying gallbladder hemorrhage vary. In one scenario, acute inflammation in the gallbladder, often triggered by factors such as gallstones, leads to necrosis and erosion of the mucosa, followed by bleeding from blood vessels in the gallbladder wall as the inflammation progresses [11,12]. Alternatively, hemorrhage may occur initially because of arteriosclerosis of blood vessels or tumors in the gallbladder wall. Bleeding or the formation of blood clots increases gallbladder pressure, resulting in ischemic changes in the wall and subsequent secondary development of cholecystitis [13]. In this case, the patient experienced abdominal pain for five days and showed elevated inflammatory markers, findings consistent with acute cholecystitis. However, the clinical course does not clarify whether cholecystitis initiated the process or resulted from hemorrhage. Both pathways remain plausible. COVID-19 likely contributed to the development of HC in this patient.

A matched-cohort study has demonstrated a significantly increased risk of bleeding events in individuals with COVID-19 [14]. Including this report, four cases of HC following COVID-19 infection have been documented (Table 2) [4-6]. While gallstones are the most common precipitating factor in cholecystitis, acalculous forms account for approximately 5%-10% of acute cholecystitis cases and are more frequently seen in critically ill or immunocompromised patients [15]. Interestingly, all cases in Table 2 were acalculous. Unlike typical cholecystitis, which often arises in the context of gallstone-related inflammation and mechanical obstruction, COVID-19-associated HC appears to emerge through alternative mechanisms.

**Table 2:** Cases of hemorrhagic cholecystitis after treatment of COVID-19 COVID-19: coronavirus disease 2019; Lap-C: laparoscopic cholecystectomy; US: ultrasound; HT: hypertension; IHD: ischemic heart disease; CKD: chronic kidney disease; T2DM: type 2 diabetes mellitus; MRCP: magnetic resonance cholangiopancreatography; AF: atrial fibrillation; CT: computed tomography

Reference	Age/sex	Anticoagulant	Comorbidity	Steroid for COVID-19	Onset of abdominal pain after diagnosis of COVID-19 (days)	Image modality	Image findings	Gallstone	Management	Complication	Outcome
Cirillo et al. [4]	79/Male	Enoxaparin 6,000 I.U. twice per day	Unclear	Yes (methylprednisolone)	7	CT with contrast	Active contrast extravasation	No	Lap-C	No	Recovered
Cochran et al. [5]	67/Male	N/A	Hepatitis C virus, moderate emphysema, HT, IHD, heart transplant	Unclear	15	CT with contrast, US	Hyperattenuating layering material with active contrast extravasation	No	Percutaneous cholecystostomy tube and antibiotics	No	Improved
Anouassi, et al. [6]	67/Male	Apixaban 5 mg twice daily	CKD, HT, T2DM	Yes (prednisolone)	21	CT (unknown about contrast), MRCP	Distended hyperattenuating gallbladder on CT, intraluminal layering, and early subacute blood products in MRCP	No	Conservative	No	Recovered
Our case	87/Male	Apixaban 5 mg daily	AF	Yes (dexamethasone)	7	CT with contrast	Active contrast extravasation	No	Lap-C	No	Recovered

All cases involved the use of corticosteroids such as methylprednisolone, prednisolone, or dexamethasone. Corticosteroids can compromise mucosal integrity, delay tissue repair, and promote vasculitis, all of which may lead to gallbladder wall fragility and hemorrhage. In addition to steroid effects, COVID-19 itself induces a systemic prothrombotic and inflammatory state. COVID-19 has been strongly associated with endothelial dysfunction, microvascular thrombosis, and hypercoagulability [16]. These mechanisms can impair gallbladder wall perfusion and predispose to both ischemia and hemorrhage. The cytokine-mediated inflammatory response further exacerbates vascular injury. The onset of abdominal pain between 7 and 21 days after COVID-19 diagnosis is consistent with a subacute, evolving vascular process rather than an immediate direct viral insult.

Taken together, these findings support the hypothesis that COVID-19 can act as a trigger for HC through a combination of vascular injury, thrombotic complications, and steroid-induced tissue fragility. The clinical course and timing are compatible with a COVID-19-related vascular mechanism leading to hemorrhage and subsequent cholecystitis.

Cholecystectomy is the primary treatment for HC, provided that the patient is fit to undergo surgery [17]. There are also reports of interventional radiology (IVR) being used to achieve hemodynamic stability in patients with hemorrhagic shock [18,19]. IVR can be valuable for stabilizing the patient and reducing the time until surgery, although it is common for gangrenous cholecystitis to develop after IVR, necessitating cholecystectomy [20]. In the present case, the patient was not in shock and underwent a laparoscopic cholecystectomy without IVR. Prompt diagnosis of HC is critical, and the choice between cholecystectomy or IVR hemostasis should be guided by the presence or absence of hemorrhagic shock.

## Conclusions

HC is a rare but life-threatening condition and its incidence is expected to increase owing the increasing use of anticoagulant therapy and its potential associations with COVID-19. Although the underlying pathogenesis remain unclear, clinicians should be aware of the potential for HC in patients with acute abdominal pain with COVID-19, particularly those receiving anticoagulants and corticosteroids. Corticosteroid use may contribute to mucosal fragility or vascular injury, potentially increasing the risk of gallbladder hemorrhage. Contrast-enhanced CT should be prioritized in suspected cases to allow for early diagnosis and timely surgical intervention. Further accumulation of case reports and systematic reviews may improve recognition and lead to the prevention of disease onset and early treatment of this critical condition.
